# Untangling the transmission dynamics of primary and secondary vectors of *Trypanosoma cruzi* in Colombia: parasite infection, feeding sources and discrete typing units

**DOI:** 10.1186/s13071-016-1907-5

**Published:** 2016-12-01

**Authors:** Carolina Hernández, Camilo Salazar, Helena Brochero, Aníbal Teherán, Luz Stella Buitrago, Mauricio Vera, Hugo Soto, Zulibeth Florez-Rivadeneira, Sussane Ardila, Gabriel Parra-Henao, Juan David Ramírez

**Affiliations:** 1 Grupo de Investigaciones Microbiológicas-UR (GIMUR), Programa de Biología, Facultad de Ciencias Naturales y Matemáticas, Universidad del Rosario, Bogotá, 111221 Colombia; 2 Estudiante Doctoral, Doctorado Ciencias biomédicas y biológicas, Universidad el Rosario, Bogotá, Colombia; 3 Biology Program, Faculty of Natural Sciences and Mathematics, Universidad del Rosario, Carrera. 24 No. 63C-69, Bogotá, DC 111221 Colombia; 4 Facultad de Ciencias Agrarias, Universidad Nacional de Colombia, Bogotá, Colombia; 5 Grupo de Investigación COMPLEXUS, Fundación Universitaria Juan N. Corpas, Bogotá, Colombia; 6 Laboratorio de Salud Pública del Meta, Villavicencio, Colombia; 7 Ministerio de Salud y protección Social, Bogotá, Colombia; 8 Laboratorio de Salud Pública del Cesar, Valledupar, Colombia; 9 Laboratorio de Salud Pública, Secretaría de Salud de La Guajira, La Guajira, Colombia; 10 Grupo de Entomología, Instituto Nacional de Salud, Bogotá, Colombia; 11 Centro de Investigación en Salud para el Trópico, Universidad Cooperativa de Colombia, Santa Marta, Colombia

**Keywords:** Chagas disease, Secondary vectors, *Trypanosoma cruzi*, DTUs, Feeding sources, Colombia

## Abstract

**Background:**

*Trypanosoma cruzi* is the causative agent of Chagas disease. Due to its genetic diversity has been classified into six Discrete Typing Units (DTUs) in association with transmission cycles. In Colombia, natural *T. cruzi* infection has been detected in 15 triatomine species. There is scarce information regarding the infection rates, DTUs and feeding preferences of secondary vectors. Therefore, the aim of this study was to determine *T. cruzi* infection rates, parasite DTU, ecotopes, insect stages, geographical location and bug feeding preferences across six different triatomine species.

**Methods:**

A total of 245 insects were collected in seven departments of Colombia. We conducted molecular detection and genotyping of *T. cruzi* with subsequent identification of food sources. The frequency of infection, DTUs, TcI genotypes and feeding sources were plotted across the six species studied. A logistic regression model risk was estimated with insects positive for *T. cruzi* according to demographic and eco-epidemiological characteristics.

**Results:**

We collected 85 specimens of *Panstrongylus geniculatus*, 77 *Rhodnius prolixus*, 37 *R. pallescens*, 34 *Triatoma maculata*, 8 *R. pictipes* and 4 *T. dimidiata*. The overall *T. cruzi* infection rate was 61.2% and presented statistical associations with the departments Meta (OR: 2.65; 95% CI: 1.69–4.17) and Guajira (OR: 2.13; 95% CI: 1.16–3.94); peridomestic ecotope (OR: 2.52: 95% CI: 1.62–3.93); the vector species *P. geniculatus* (OR: 2.40; 95% CI: 1.51–3.82) and *T. maculata* (OR: 2.09; 95% CI: 1.02–4.29); females (OR: 2.05; 95% CI: 1.39–3.04) and feeding on opossum (OR: 3.15; 95% CI: 1.85–11.69) and human blood (OR: 1.55; 95% CI: 1.07–2.24). Regarding the DTUs, we observed TcI (67.3%), TcII (6.7%), TcIII (8.7%), TcIV (4.0%) and TcV (6.0%). Across the samples typed as TcI, we detected TcIDom (19%) and sylvatic TcI (75%). The frequencies of feeding sources were 59.4% (human blood); 11.2% (hen); 9.6% (bat); 5.6% (opossum); 5.1% (mouse); 4.1% (dog); 3.0% (rodent); 1.0% (armadillo); and 1.0% (cow).

**Conclusions:**

New scenarios of *T. cruzi* transmission caused by secondary and sylvatic vectors are considered. The findings of sylvatic DTUs from bugs collected in domestic and peridomestic ecotopes confirms the emerging transmission scenarios in Colombia.

**Electronic supplementary material:**

The online version of this article (doi:10.1186/s13071-016-1907-5) contains supplementary material, which is available to authorized users.

## Background

Chagas disease caused by the protozoan parasite *Trypanosoma cruzi*, affects about six million people in Latin America. The main transmission mechanism is by insect vectors (stercoralian route). The insects responsible for vector transmission belong to the subfamily Triatominae (Hemiptera: Reduviidae), composed by approximately 140 species of 5 tribes [1]. The natural habitats of triatomines include palm trees, tree holes, cracks in rocks, small caves and other animal shelters [2]. The main vectors of *T. cruzi* in the Southern Cone countries are *Triatoma infestans*, *Triatoma brasiliensis* and *Panstrogylus megistus*; *Rhodnius prolixus* and *Triatoma dimidiata* in the Andean region and parts of Central America, and *Triatoma dimidiata* and *T. barberi* in Mexico [3]. A total of 26 species have been reported in Colombia; of these 15 have been shown to be naturally infected with *T. cruzi* [4]*.* Thus, *R. prolixus* and *T. dimidiata* are considered primary vectors, whereas *P. geniculatus*, *T. maculata*, *R. pictipes* and *R. pallescens* are considered secondary vectors, among others that have been found naturally infected with *T. cruzi*. In Colombia, there are approximately 436,000 people infected with *T. cruzi*, with an annual incidence of 5,250 vector-borne cases per population [5]. In addition, some studies revealed that species at greatest risk of transmission are *R. prolixus*, *T. dimidiata*, *T. maculata* and *T. venosa* [4]*.* However, vector control programs have focused on domiciled species as *R. prolixus* and *T. dimidiata*.

*Trypanosoma cruzi* transmission mostly occurs in three epidemiological cycles: sylvatic (enzootic), domestic and peridomestic, where the parasite circulates among triatomines, mammal reservoirs and human hosts. Around 180 sylvatic and synanthropic species of mammal have been described to date, which act as reservoirs of *T. cruzi* inhabiting places near human settlements [6]. *Trypanosoma cruzi* exhibits remarkable genetic diversity and has been classified by international consensus in six Discrete Typing Units (DTUs) (TcI-TcVI), plus a new genotype associated to anthropogenic bats (TcBat) [7]. The DTUs present associations with transmission cycles, geography, vector species and clinical manifestation to some extent [7–12]. The DTU with the broadest geographical distribution is TcI and due to its high genetic diversity has been divided into two genotypes, associated to domestic and sylvatic foci (TcIDom and Sylvatic TcI, respectively) [13–16].

Current molecular tools have improved the detection and genotyping of the parasite in the vectors as well as the identification of the vectors’ feeding sources involved in the transmission of the parasite. However, in Colombia there is scarce information about the relationship between different vector species, *T. cruzi* infection and eco-epidemiological aspects of the transmission cycles. Control programs in this country have focused on domiciled vectors while epidemiological and scientific research has been restricted to the Caribbean region. Therefore, the study of vectors in other areas of the country with a holistic perspective that considers *T. cruzi* infection rates, genotyping and feeding sources will help to identify the hosts and understand the dynamics of parasite transmission. This information will be useful to generate vector control strategies and accurate surveillance of Chagas disease. Thus, the objective of this study was to apply this holistic perspective to six triatomine species collected in different transmission cycles from seven departments in Colombia.

## Methods

### Study area and collection of triatomines

**Fig. 1:**
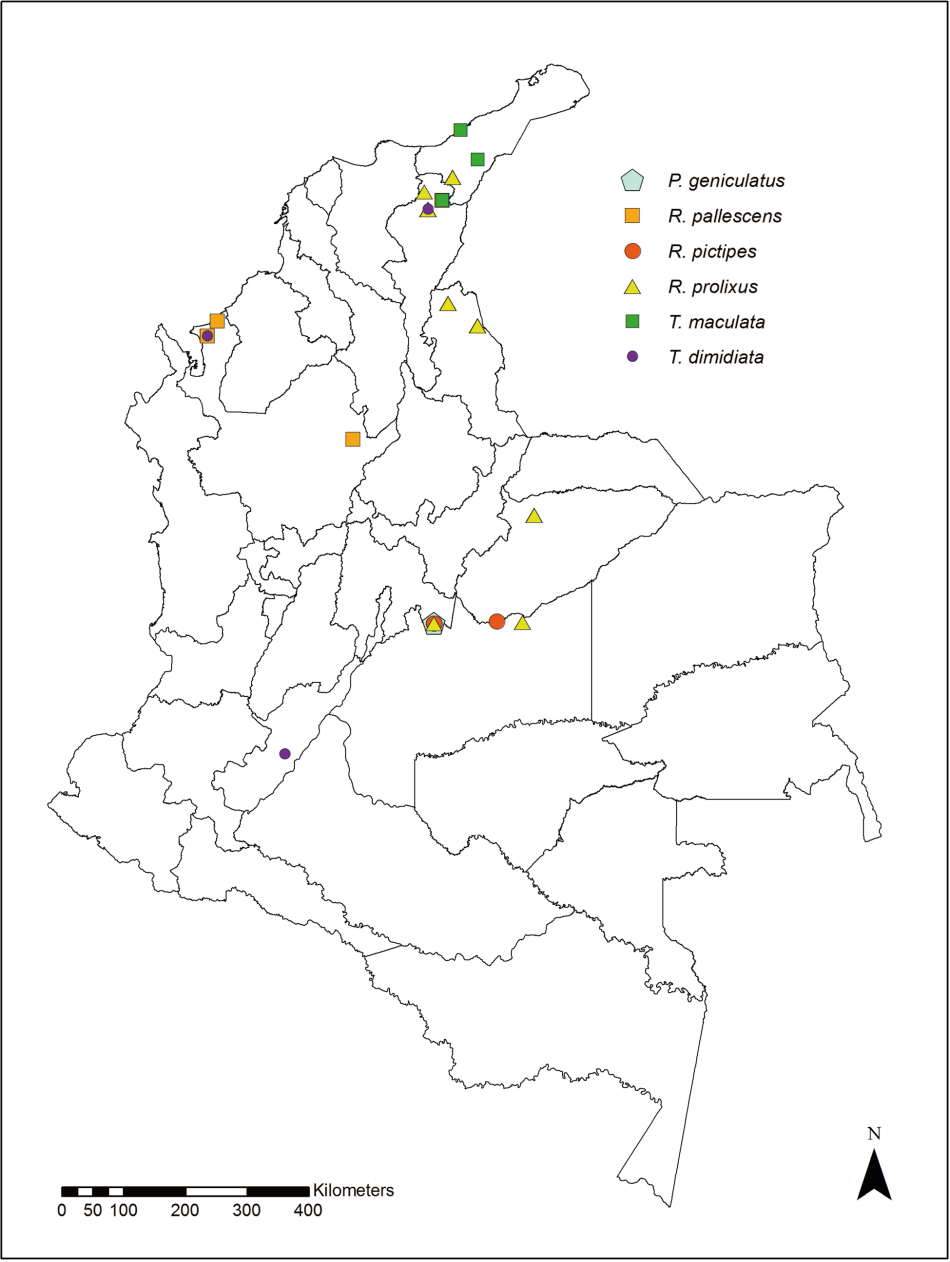
Geographical distribution of 245 triatomines collected across Colombia and included in this study

**Fig. 2:**
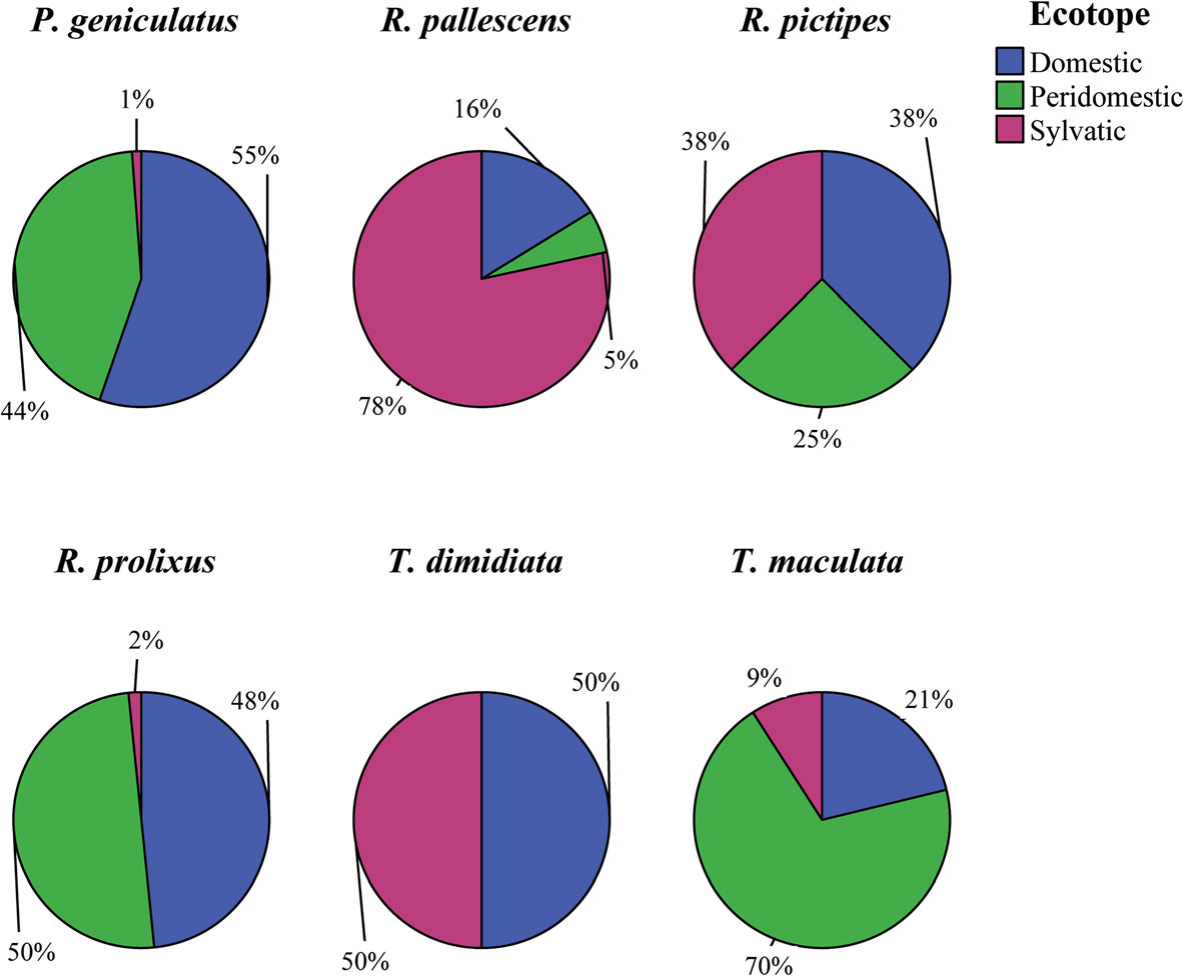
Frequency of ecotopes for the species studied. Samples were collected in sylvatic, peridomestic and domestic ecotopes

A total of 245 specimens corresponding to six species (85 *P. geniculatus*, 77 *R. prolixus*, 37 *R. pallescens*, 34 *T. maculata*, 8 *R. pictipes* and 4 *T. dimidiata*) were collected in the departments Guajira, Antioquia, Cesar, Norte de Santander, Meta, Casanare and Huila (Fig. 1; Additional file 1: Table S1). Insect capture in the sylvatic cycle was performed using two techniques: manual search and modified Noireau baited chicken traps in palms and distant to housing areas. Additionally, insects inside houses and in the peridomestic ecotope were collected. All insects were stored in separate jars, including a description of the site of collection and georeferenced using GPS. The insects were identified using taxonomic keys and stored in 100% ethanol until processing [17]. All of the specimens except *T. dimidiata* were collected from domestic, peridomestic and sylvatic cycles of transmission (Additional file 1: Table S1; Fig. 2).

### Molecular detection of *T. cruzi* and genotyping

DNA extraction of the complete body of each insect was conducted using the ZR Tissue & Insect miniprep DNA Zymo ™ kit (Zymo Research, Irvine, USA), then endpoint qPCR was performed for detecting the satellite DNA of *T. cruzi* using primers cruzi1 (5′-AST CGG CTG ATC GTT TTC-3′), cruzi2 (5′-AAT TCC TCC AAG CAG CGG ATA-3′) and cruzi3 probe (FAM-CAC ACA CTG GAC ACC AA-NFQ-MGB) using the conditions previously reported [18]. In the interpretation, since no *T. cruzi* DNA quantitation was performed, it is interpreted as positive DNA amplification in Cts < 38 and negative amplification as absence. 12S subunit ribosomal gene of triatomines was used as internal amplification control under the conditions and primers previously described [19]. Subsequently, the insects with positive results by qPCR were submitted to kinetoplast DNA amplification using primers 121 (5′-AAA TAA TGT ACG GGK GAG ATG CAT GA-3′) and 122 (5′-GGT TCG ATT GGG GTT GGT GTA ATA TA-3′) to discriminate *T. cruzi* and *T. rangeli* infections as previously reported [20]. DNA from strains MHOM/CO/01/DA and RHO/CO/82/Durán were used as positive controls of *T. cruzi* and *T. rangeli*, respectively. The identification of DTUs was accomplished by conventional PCR using the SL-IR, 18 s, 24 s and A10 targets as previously described [11, 21, 22]. We employed reference strains from each DTU as follows: TcIDom (DA), TcISylvatic (GC), TcII (Y), TcIII (CM17), TcIV (YLY), TcV (Tulahuen) and TcVI (CLBrener).

### Molecular characterization of blood sources

All the 245 specimens were submitted to identification of feeding preferences by PCR-HRM (Polymerase chain reaction-High resolution melting) as previously reported [23]. Also, the feeding preferences were corroborated by direct sequencing of *cytb* using the primers CytbFw (5′-CCC CTC AGA ATG ATA TTT GTC CTC A-3′) and CytbRv (5′-CCA TCC AAC ATC TCA GCA TGA TGA AA-3′). The resulting sequences were edited in MEGA 6.0 [24] and submitted to BLASTn similarity search. This methodology allows the detection of 14 host species involved in the epidemiological cycles of Chagas disease as previously reported [23].

### Statistical analyses

We calculated the frequency of *T. cruzi* infection, DTUs, TcI genotypes and feeding preferences across species and ecotopes (transmission cycles). To establish the association between the variables, Chi-square test was implemented with Monte Carlo adjustment with 10,000 simulations and G-test including pairwise comparison. G-test was not applied in cases where the contingency table had zeros (Additional file 2: Table S2). Additionally, using an unconditional logistic regression, without including the intercept, the risk for infection with *T. cruzi* was estimated (OR, 95% IC) according to the demographic and ecoepidemiological characteristics using EpiInfo V.3.5.4 software and R package version 3.3.1.1. Statistical significance was established with a *P*-value < 0.05.

## Results

### *Trypanosoma cruzi* detection in six species of triatomines

**Fig. 3:**
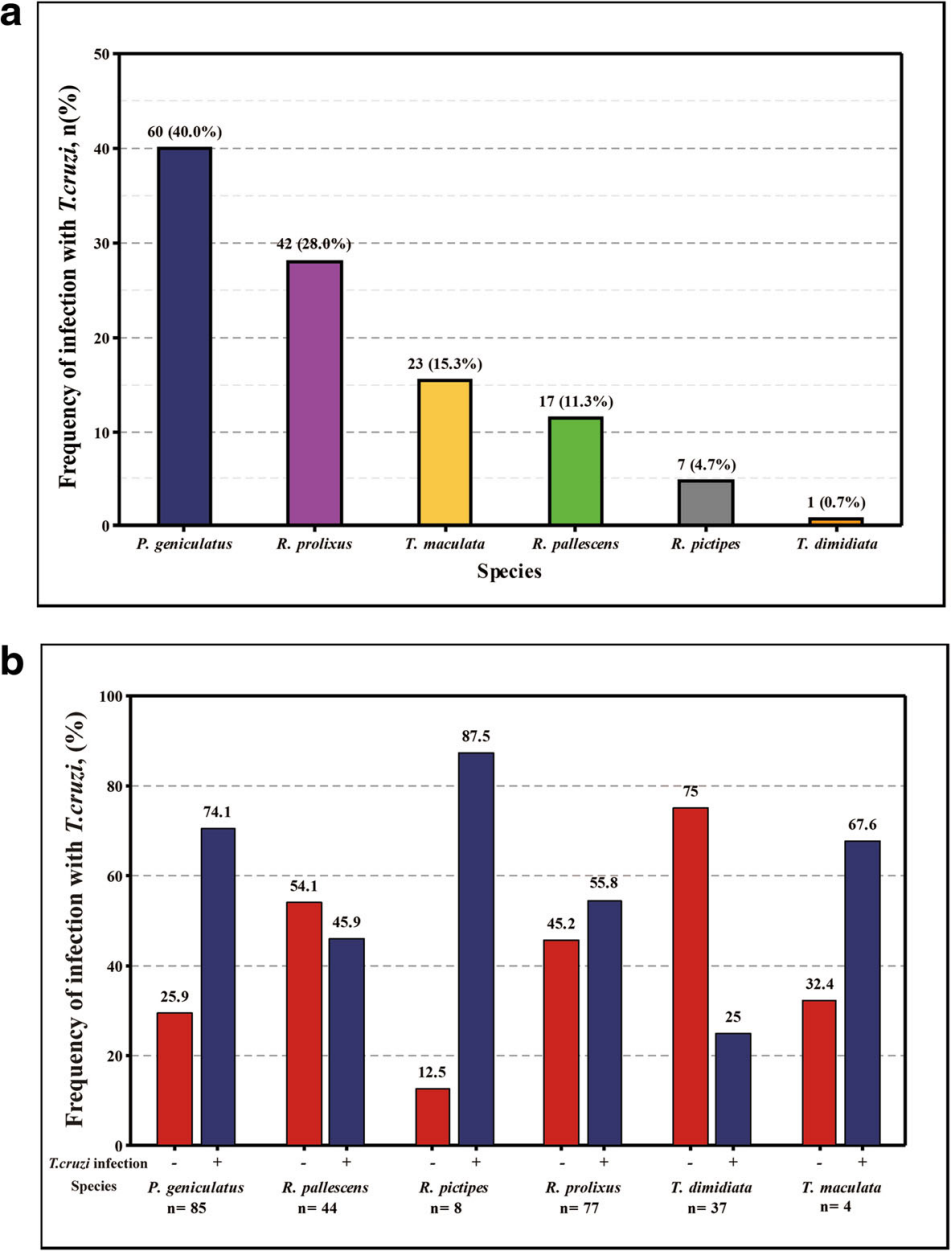
Frequency of infection with *T. cruzi* in the triatomines collected. **a** Frequency of infection with *T. cruzi* in all samples. **b** Frequency of insects negative (–) and positive (+) for *T. cruzi* in all species collected

The information regarding the frequency of infection with *T. cruzi* by geographical location, ecotope and insect stage is shown in Table 1. The overall *T. cruzi* infection rate was 61.2% (*n* = 150). The species with the highest percentage of infection with *T. cruzi* was *P. geniculatus*, followed by *R. prolixus* and *T. maculata* (Fig. 3a). In addition, one *R. prolixus* was positive for *T. rangeli*.

**Table 1:** Frequency of infection with *T. cruzi* by geographical location, ecotope and insect stage

Department	*n*	% of infection with *T. cruzi*	95% CI
Meta	69	46.0	38.0–53.9
Guajira	32	21.3	14.7–27.8
Cesar	24	16.0	9.5–21.1
Antioquía	12	8.0	3.6–12.3
Norte de Santander	9	6.0	2.2–9.8
Casanare	3	2.0	0.4–5.7
Huila	1	0.7	0.02–3.6
Ecotope			
Peridomestic	68	45.3	39.7–56.1
Domestic	52	34.7	28.7–44.5
Sylvatic	22	14.7	9.5–21.4
Stage			
Female	76	50.7	44.3–60.5
Male	43	28.7	22.2–37.1
Nymph	26	17.3	11.7–24.2

*Abbreviation*: n, number of positive samples

The frequency of *T. cruzi* infection within each species was as follows: *T. maculata*: 67.6% (23/34); *P. geniculatus*: 70.6% (60/85); *R. pallescens*: 45.9% (17/37); *R. prolixus*: 55.8% (43/77); *R. pictipes*: 87.5% (7/8); and *T. dimidiata*: 25.0% (1/4) as shown in Fig. 3b. Association between species and the infection with *T. cruzi* was found (*χ* ^2^ = 13.35, *df* = 5, *P* = 0.0171; G-test, *G* = 13.33, *P* = 0.0175), therefore statistical analysis was performed within each species using logistic regression (Table 2) and by Chi-square and G-test analysis (Additional file 2: Table S2; Additional file 3: Table S3). The species that showed association with the infection by *T. cruzi* were *P. geniculatus* and *T. maculata. Panstrongylus geniculatus* showed association with *T. cruzi* infection by the three statistical analyses. G-test revealed significant pairwise differences specifically with *R. pallescens* and *R. prolixus. Trypanosoma maculata* showed association with positive rate by Chi-square and logistic regression. Additionally, *R. pictipes* showed association only by Chi-square analysis. However, this is because the sample size is very small, which is reflected in the wide confidence interval in the logistic regression that invalidates the significant OR (Table 2; Additional file 2: Table S2; Additional file 3: Table S3). Furthermore, the association between species and ecotopes was statistically significant (*χ* ^2^ = 142.82, *df* = 8, *P* < 0.0001; G-test, *G* = 123.17, *P* < 0.0001). By analyzing G-pairwise comparison test, significant differences were observed among all species except for *R. prolixus* compared to *P. geniculatus* and *R. pictipes* compared to *R. pallescens* (Additional file 4: Table S4).

**Table 2:** Variables associated with infection with *T. cruzi* across the insect vectors studied

Characteristic		Infection with *T. cruzi*	
	Odds Ratio	95% CI	*P*-value
Species			
*P. geniculatus*	**2.40**	**1.51–3.82**	**0.0001**
*T. maculata*	**2.09**	**1.02–4.29**	**0.044**
*R. pictipes*	7.0	0.86**–**56–8	0.068
*R. prolixus*	1.20	0.76–1.88	0.420
*R. pallescens*	0.85	0.44–1.62	0.622
*T. dimidiata*	0.33	0.03–3.20	0.341
Department			
Casanare	1.50	0.25–8.98	0.657
Cesar	1.00	0.56–1.78	1.000
Guajira	**2.13**	**1.16–3.94**	**0.016**
Huila	1.00	0.06–15.99	1.000
Meta	**2.65**	**1.69–4.17**	**0.0001**
Norte de Santander	0.82	0.34–1.97	0.655
Feeding source			
Armadillo	1.00	0.06–15.99	1.000
Canine^a^	1473120.79	7.00–>1.0e^12^	0.957
Opossum	**3.15**	**1.85–11.69**	**0.087**
Hen	0.60	6.26–1.40	0.235
Human	**1.55**	**1.07–2.24**	**0.022**
Bat	1.47	0.60–3.60	0.400
Mouse	1.50	5.42–5.32	0.530
Rodent	2.00	0.37–10.92	0.424
Ecotopes			
Sylvatic	1.38	0.72–2.62	0.332
Domestic	1.24	0.82–1.86	0.303
Peridomestic	**2.52**	**5.61–3.93**	**0.0001**
Stage			
Female	**2.05**	**1.39–3.04**	**0.0001**
Male	1.39	0.87–2.20	0.165
Nymph	1.18	0.67–2.09	0.564

^a^The result is due to the low sample size

Significant values are indicated in bold

Regarding the infection with *T. cruzi* and the variables listed in Table 1, statistically significant association was evident with geographical location (*χ* ^2^ = 16., *df* = 6, *P* = 0.0066; G-test, *G* = 16.93, *P* = 0.0095). As for the geographical location, two departments (Guajira and Meta) exhibited a positive association with *T. cruzi* (Table 2). In both cases, these departments showed differences with departments Antioquia and Cesar; additionally, there were differences between Meta and Norte de Santander (Additional file 5: Table S5). No association with infection with *T. cruzi* was found regarding ecotope (*χ* ^2^ = 5.75, *df* = 2, *P* = 0.0542; G-test, *G* = 5.83, *P* = 0.0539) and life stage (*χ* ^2^ = 2.051, *df* = 2, *P* = 0.3586; G-test, *G* = 2.050, *P* = 0.3864). However, logistic regression detected association between the peridomestic ecotope, female stage and *T. cruzi* infection (Table 2).

### Feeding preferences across the six species of triatomines

**Fig. 4:**
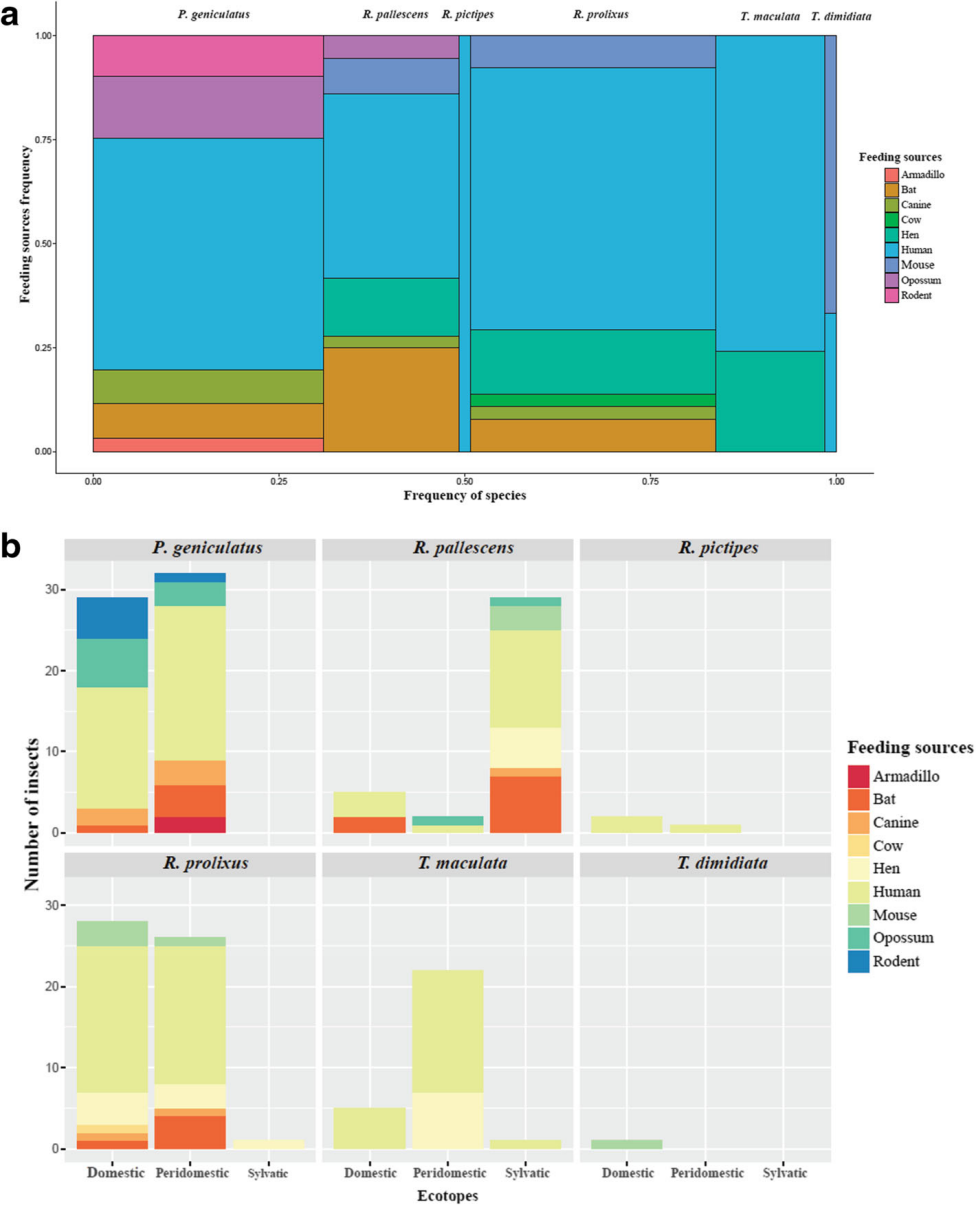
Feeding sources in triatomines collected. **a** Feeding preferences across the six species of vector collected. **b** Feeding sources in species of triatomines collected by ecotope

A total of 9 feeding sources were detected in 197 insects corresponding to six species. The frequencies were as follows: human blood: 59.4% (117); hen: 11.2% (22); bat: 9.6% (19); opossum: 5.6% (11); mouse: 5.1% (10); dog: 4.1% (8); rodent: 3.0% (6); armadillo: 1.0% (2); and cow: 1.0% (2). Association of the feeding sources and *T. cruzi* infection was observed specifically with human blood and opossum (*χ* ^2^ = 12.917, *df* = 8, *P* = 0.1002) (Table 2). Additionally, we evaluated the feeding preferences across 122 insects that were *T. cruzi-*positive and found human blood in 58.2% (71), hen in 7.4% (9), bat in 9.8% (12), opossum in 7.4% (9), mouse in 4.9% (6), dog in 6.6% (8), rodent in 3.3% (4), armadillo in 0.8% (1) and cow in 1.6% (2). Regarding the feeding preferences by species, statistically significant association was observed (*χ* ^2^ = 99.56, *df* = 40, *P* = 0.0015). The insect vectors with the greatest variety of feeding sources were *P. geniculatus*, *R. prolixus* and *R. pallescens* (Fig. 4a). When feeding preferences were evaluated by ecotope, statistically significant association was found (*χ* ^2^ = 25.74, *df* = 16, *P* = 0.0468) and the most common sources were human, hen, bat and opossum (Fig. 4b). Finally, the species positive for *T. cruzi* and blood-feeding on humans are shown in Table 3.

**Table 3:** Frequency of infection with *T. cruzi* and human blood-feeding within each species

Species	*n*	*N*	Frequency of infection with *T. cruzi* (%)	Frequency of infection with *T. cruzi* and human blood-feeding (%)
*P. geniculatus*	60	85	70.59	27.06
*T. maculata*	23	34	67.65	47.06
*R. pallescens*	17	37	45.95	16.22
*R. prolixus*	43	77	55.84	32.47

*Abbreviations*: *n* Number of infected with *T. cruzi*, *N* Total number

### *Trypanosoma cruzi* DTUs and TcI genotypes

**Fig. 5:**
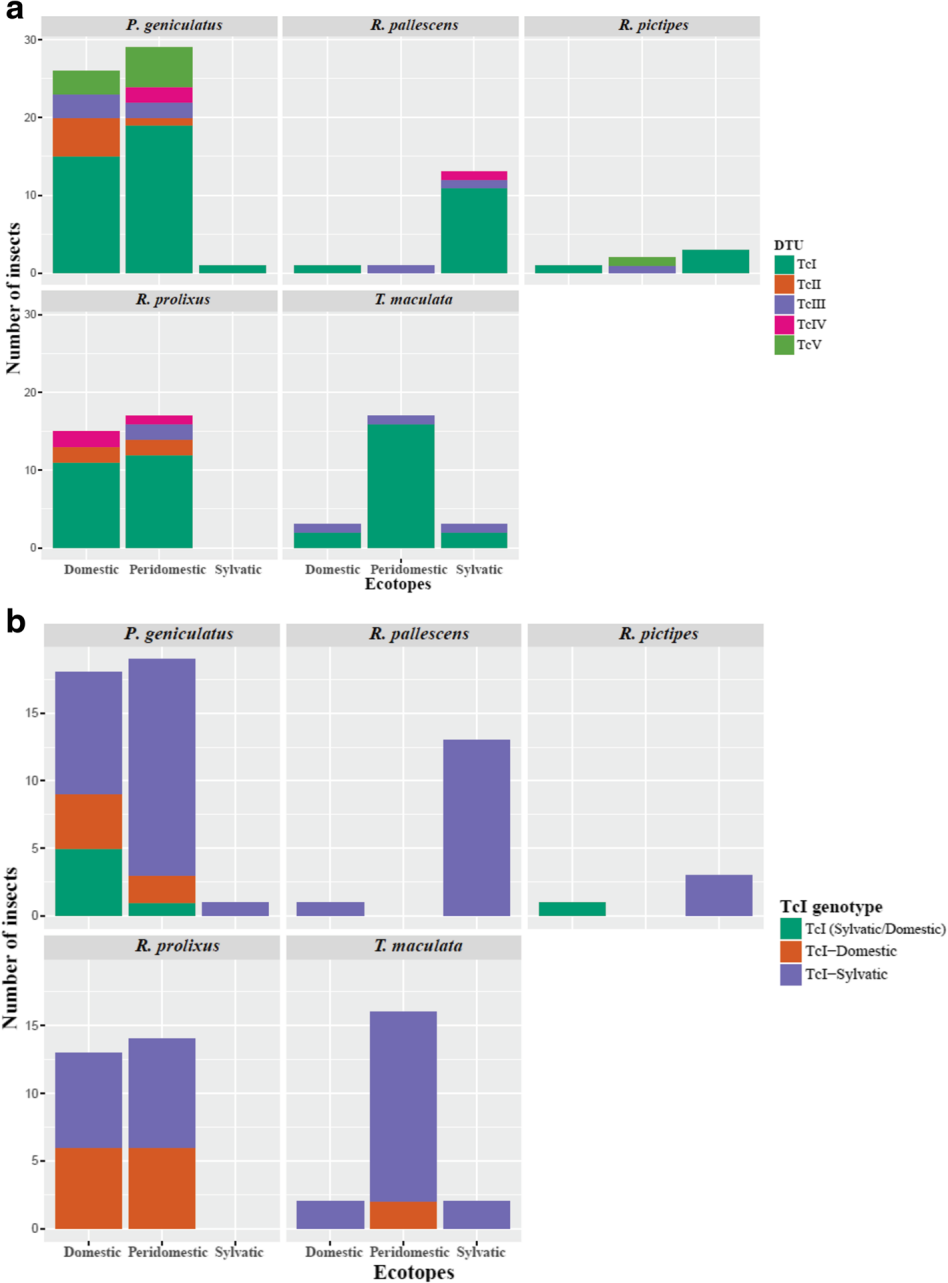
Distribution of *T. cruzi* (DTUs) and TcI genotypes according to species and ecotope. **a** Distribution of DTUs (TcI-TcVI) in the six species collected and ecotopes. **b** Distribution of TcI genotypes (TcIDom, TcI sylvatic and TcI sylvatic/TcIDom across species and ecotopes

DTUs and TcI genotypes characterization was performed on 149 samples that were positive for *T. cruzi*. The frequencies were analyzed according to species and ecotope (Fig. 5a). We found cases of single and mixed infections observing TcI in 67.8% (101), TcII in 6.7% (10), TcIII in 8.7% (13), TcIV in 4.0% (6), TcV in 6.0% (9) and mixed infections in 6.7% (10/149). No association was found between DTUs and species (*χ* ^2^ = 22.29, *df* = 20, *P* = 0.3419), feeding sources (FD: 32, *χ* ^2^ = 34.08, df = 32, *P* = 0.6476) and/or ecotopes (*χ* ^2^ = 13.88, *df* = 8, *P* = 0.1733).

Regarding the TcI genotypes, we detected TcIDom in 19.0% (19/100), sylvatic TcI in 75.0% (75/100) and TcIDom/TcIsylvatic in 6% (6/100) of the mixed infections corresponded to TcI sylvatic + TcII, TcIDom + TcII, TcIsylvatic + TcIII, TcIsylvatic + TcIII + TcIV, TcIsylvatic + TcIV and TcIsylvatic + TcV. We determined the infecting DTU and TcI genotypes discriminated by the species and ecotopes (Fig. 5b). Also, association between the TcI genotypes and the feeding sources (*χ* ^2^ = 94.21, *df* = 16, *P* = 0.0137), ecotopes (*χ* ^2^ = 17.32, *df* = 4, *P* = 0.0013) and species (*χ* ^2^ = 29.46, *df* = 10, *P* = 0.0049).

## Discussion

Triatomine species collected in this study with the exception of *R. prolixus* and *T. dimidiata* are considered sylvatic and secondary vectors. Nevertheless, they were mainly collected in domestic ecotopes (Fig. 2), and there was an association between the ecotopes and species; and the main dietary source was human blood mainly in the domestic ecotope. Feeding sources with sylvatic reservoirs blood was the lowest in our dataset. These findings reflect the intrusion of vectors from sylvatic habitats to domestic habitats and their adaptation to the available feeding sources.

The high percentage of infection with *T. cruzi* and its relationship to the specific tested variables (peridomestic ecotope, feeding with human and opossum blood), together with the occurrence of sylvatic DTUs (sylvatic TcI and TcIII); *P. geniculatus* and *T. maculata* with high percentages of infection and feeding with human blood. They all suggest the existence of possible new transmission scenarios caused by intrusion of secondary vectors (mainly *P. geniculatus* and *T. maculata* in Meta and Guajira departments, respectively). The association of TcI genotypes with ecotopes, feeding sources and species is relevant given that the higher frequencies corresponded to sylvatic TcI, domestic ecotopes, feeding sources of humans, domestic and sylvatic animals. Therefore, since the statistically significant associations are the evidence of parasite population’s movement from sylvatic to “domestic” populations with sylvatic strains is confirmed. This is of paramount relevance due to the impact of sylvatic *T. cruzi* in the acute phase and outbreaks of oral transmission. Additionally, statistically significant association between food sources (human and opossum) and *T. cruzi* infection reaffirms the potential of *D. marsupialis* as an important reservoir of the parasite [25, 26].

Our results are in accordance with other studies conducted in the Caribbean region of Colombia, which showed that secondary vectors play an important role in the different epidemiological transmission cycles of *T. cruzi*. These studies have shown that the frequency of patients with positive serology in the presence of sylvatic vectors is similar to the frequency in the presence of domestic vectors [4, 27]. Furthermore, our findings reinforce the role of *R. prolixus* as a domestic vector in Colombia given that among the triatomine species collected in this study, *R. prolixus* had the highest frequency of feeding with human blood and at the same time showed high rate of *T. cruzi* infection (33.8%). The frequency of *R. prolixus* specimens collected in domestic habitats was 98.4% (Fig. 3a). The feeding sources of *R. prolixus* were mainly humans and domestic animals (Fig. 4a) and the DTUs detected were primarily associated with domestic cycles (TcI and TcII), and mostly infected with TcIDom (Fig. 5). These findings must be a support for the vector control programs in the country. Mainly, because most of the efforts have been focused on domestic vectors such as *R. prolixus* and *T. dimidiata* and our findings evidenced the potential risk of *T. cruzi* transmission by secondary vectors. Therefore, monitoring and control strategies specifically designed for sylvatic vectors are required in Colombia [28].

Surprisingly, we detected TcV in specimens of *P. geniculatus* and *R. pictipes* (sylvatic vectors). This DTU has been reported in domestic cycles from southern Latin-American countries [7, 29]. However, insects infected with TcV have been reported in domestic habitats in Colombia including some reports of human infections [10, 30]. TcV associated with sylvatic cycles has been reported with a frequency of 3.5% [29]. Consistent with our findings of TcV, a recent study [31] reported the presence of TcV in Colombian isolates obtained from *P. geniculatus*, *R. prolixus*, *T. venosa* and armadillos, using high-resolution markers: MLST, MLMT and ten mitochondrial markers. Messenger et al. [31] have also shown that the Colombian TcV isolates are due to migration processes from Southern Cone countries and not to local hybridization processes.

*Triatoma maculata* has a wide geographical distribution in Colombia. We observed a high invasion of domestic ecotopes by this species (Fig. 2), consistent with other studies in Colombia, Brazil and Venezuela where this species even presents morphological and genetic changes across individuals collected in domestic ecotopes [26, 27, 32–34]. *Triatoma maculata* has not been included in the vector control programs because its diet is mainly composed of bird blood [32, 35–37] and some studies have reported low frequency of infection in Brazil and Venezuela [36, 38]. By contrast, herein the frequency of *T. cruzi* infection was 67.6% and the percentage of feeding with human blood was 75.0% with the presence of TcIDom in some specimens collected in peridomiciliary habitats. Recent studies in Colombia and Venezuela have revealed infection frequencies between 38.0 and 75.0% and the presence of “TcIb” genotype that is associated with the peridomestic cycle [26, 27, 33, 34, 39, 40]. Regarding the DTUs herein detected, most of the specimens were infected with TcI and TcIII (sylvatic DTUs) and domestic specimens harbored TcIII suggesting how *T. maculata* can connect domestic and sylvatic transmision cycles (Fig. 4). Our results and previous reports highlight the relevance of *T. maculata* as a potential vector in Colombia suggesting the need to prioritize this species in vector control programs, and additionally, to be cautious about the potential risk of domiciliation that this species may have.

*Panstrongylus geniculatus* is the most widely distributed in Latin America species of the genus *Panstrongylus* [41]. In Colombia this species has the widest geographical distribution and is recorded in 25 departments including the Department of Meta [4]. In this study, most of the specimens were collected from the domestic ecotope (Fig. 2; Additional file 3: Table S3). *Panstrongylus geniculatus* is considered a sylvatic vector that inhabits in burrows primarily associated with armadillos, opossums, rodents and bats [41]. Accordingly, there are several reports in Latin America and Colombia demonstrating the intrusion of adult specimens of *P. geniculatus* in domestic habitats [42–45] and also findings of different nymphal stages in human dwellings mainly in Brazil, Venezuela and Colombia (Amalfi, Antioquia) [46–50]. Colonization in domestic habitats may be due to changes generated by housing construction and alteration of the ecosystems in the municipalities analyzed and/or by the attraction generated by the artificial light [51].

Our results showed *P. geniculatus* ranking first in *T. cruzi* infection (Fig. 3a; Table 2; Additional file 3: Table S3), similar to previous reports in Brazil, Venezuela and the Colombian Caribbean region [40, 46, 48, 49]. We detected five DTUs in this species (TcI-TcV) that is in accordance with previous reports in Brazil, Venezuela and the Colombian Orinoco, and showing the interesting permissivity of this species facilitating the transmission of a wide variety of DTUs [10, 46, 48, 51]. In contrast with other reports, we report a wide range of feeding sources with mammals of different epidemiological cycles and human blood. These results demonstrate the strong adaptive ability to different food sources and possibly explaining the high rate of infection and variety of DTUs. Human and canine blood constitute an important food source for *P. geniculatus* and similar to that observed in Venezuela, where also ten outbreaks of *T. cruzi* oral transmission involving this species were reported [48, 49, 52]. A small quantity of *P. geniculatus* fed on sylvatic sources (opossums, rodents, bats and armadillos) explaining the detection of sylvatic DTUs (TcI sylvatic, TcIII and TcIV) across our dataset [2, 41, 53] (Fig. 5a). All these findings could be a signal of an intrusive process from sylvatic ecotopes to domestic ones facilitated by adaptation, occurring in parallel in Colombia, Venezuela and Brazil [42–45], even accompanied by morphological changes in insects [54, 55]. This pattern of ecotope intrusion explains the incrimination of *P. geniculatus* in oral outbreaks in Colombia [28, 56]; it is mandatory that surveillance strategies are deployed not only to avoid incrimination but also a possible domiciliation process.

Another sylvatic species is *R. pallescens*, given that its habitats are tree palms of *Attalea butyracea* and the invasion in domestic ecotopes is seen through intrusion [2, 57–60]. Herein, insects were found in the three ecotopes and most specimens (both nymphs and adults) were collected in sylvatic habitats mainly in wine (*A. butyracea*) and oil (*Elaeis guineensis*) palm trees, while 21.6% (adults) were collected in domestic habitats. This is consistent with previous studies in Colombia [27, 57, 58, 60, 61] and Panama [40, 58, 62–65], where the percentage of *T. cruzi* infection is similar to our results. Moreover, it was observed that the main feeding source was human blood across the three ecotopes. This might be due to agriculture at the collection sites, where palms are nearby to homes and this can facilitate contact between the vectors and human hosts. We report that *R. pallescens* also fed on different mammals such as *D. marsupialis* in the peridomicile habitat and bats, mice and dogs in the sylvatic ecotope, showing an interplay between peridomestic and sylvatic cycles [59, 62–64, 66, 67]. Finally, in the domestic ecotope the sylvatic TcI was only detected, showing that in fact, the specimens of *R. pallescens* might correspond to intrusions in homes from the sylvatic habitat. However, in contrast Cantillo et al. [58], detected “TcIb” in specimens of *R. pallescens* collected in domestic habitats, confirming that in Colombia there is a risk of *R. pallescens* intrusion supported by the high presence blood-feeding on humans and *T. cruzi* infection.

## Conclusions

To the best of our knowledge, we conducted the first robust study sampling secondary vectors of *T. cruzi* in Colombia from different locations of the country. We used a broad variety of techniques to detect *T. cruzi* infection, DTUs, TcI genotypes and feeding sources that allowed us to understand the transmission dynamics in secondary vectors such as *P. geniculatus*, *T. maculata* and *R. pallescens*. Our findings reinforce the epidemiological relevance of these species and highlight the need to include them in the vector control programmes as well as in the entomological surveillance systems. Most of the secondary insects captured harbored sylvatic DTUs and fed on human blood highlighting the importance of these species. Our results demonstrate the need of the government to invest on the control of them in the light of their effort to interrupt *T. cruzi* transmission in Colombia.

## Additional files

Additional file 1: Table S1: Geographical coordinates and ecotopes in vectors collected. (DOC 376 kb)
Additional file 2: Table S2:
*T. cruzi* infection rates in the triatomine species studied. (DOCX 16 kb)
Additional file 3: Table S3: Pairwise G-test (*T. cruzi* infection rates by species). (DOCX 13 kb)
Additional file 4: Table S4: Pairwise G-test (Species *vs* Ecotopes). (DOCX 13 kb)
Additional file 5: Table S5: Pairwise G-test (*T. cruzi* infection rates *vs* Geographical location). (DOCX 14 kb)

## Abbreviations

DTU: Discrete typing unit
OR: Odds ratio
PCR-HRM: Polymerase chain reaction-high resolution melting
qPCR: Quantitative polymerase chain reaction
