# Supplementary material for: Untangling the transmission dynamics of primary and secondary vectors of Trypanosoma cruzi in Colombia: parasite infection, feeding sources and discrete typing units
Source: Parasit Vectors. 2016 Dec 1;9:620. doi: 10.1186/s13071-016-1907-5 (PMC5131512; doi:10.1186/s13071-016-1907-5)
Supplement: Additional file 1: Table S1: — Geographical coordinates and ecotopes in vectors collected. (DOC 376 kb) [file 13071_2016_1907_MOESM1_ESM.doc]

**Table S1. Geographic coordinates and ecotopes in vectors collected**

| **Number** | **Coordinates** | **Location** | **Species** | **Ecotopes** |
| --- | --- | --- | --- | --- |
| **1** | 11.49821,-72.98294 | Guajira | ***T. maculata*** | Peridomestic |
| **2** | 11.49821,-72.98294 | Guajira | ***T. maculata*** | Peridomestic |
| **3** | 11.49821,-72.98294 | Guajira | ***T. maculata*** | Peridomestic |
| **4** | 11.49821,-72.98294 | Guajira | ***T. maculata*** | Peridomestic |
| **5** | 11.49821,-72.98294 | Guajira | ***T. maculata*** | Peridomestic |
| **6** | 11.49821,-72.98294 | Guajira | ***T. maculata*** | Peridomestic |
| **7** | 11.49821,-72.98294 | Guajira | ***T. maculata*** | Peridomestic |
| **8** | 11.49821,-72.98294 | Guajira | ***T. maculata*** | Peridomestic |
| **9** | 11.49821,-72.98294 | Guajira | ***T. maculata*** | Peridomestic |
| **10** | 11.49821,-72.98294 | Guajira | ***T. maculata*** | Domestic |
| **11** | 11.066486,-72.737381 | Guajira | ***T. maculata*** | Domestic |
| **12** | 11.066486,-72.737381 | Guajira | ***T. maculata*** | Sylvatic |
| **13** | 11.066486,-72.737381 | Guajira | ***T. maculata*** | Sylvatic |
| **14** | 11.066486,-72.737381 | Guajira | ***T. maculata*** | Domestic |
| **15** | 11.066486,-72.737381 | Guajira | ***T. maculata*** | Peridomestic |
| **16** | 11.066486,-72.737381 | Guajira | ***T. maculata*** | Peridomestic |
| **17** | 11.066486,-72.737381 | Guajira | ***T. maculata*** | Peridomestic |
| **18** | 11.066486,-72.737381 | Guajira | ***T. maculata*** | Peridomestic |
| **19** | 11.066486,-72.737381 | Guajira | ***T. maculata*** | Peridomestic |
| **20** | 11.066486,-72.737381 | Guajira | ***T. maculata*** | Peridomestic |
| **21** | 11.066486,-72.737381 | Guajira | ***T. maculata*** | Peridomestic |
| **22** | 11.066486,-72.737381 | Guajira | ***T. maculata*** | Domestic |
| **23** | 11.066486,-72.737381 | Guajira | ***T. maculata*** | Domestic |
| **24** | 11.066486,-72.737381 | Guajira | ***T. maculata*** | Domestic |
| **25** | 11.066486,-72.737381 | Guajira | ***T. maculata*** | Domestic |
| **26** | 4.29457,-73.373255 | Meta | ***R.pictipes*** | Sylvatic |
| **27** | 4.29457,-73.373255 | Meta | ***R.pictipes*** | Sylvatic |
| **28** | 4.29457,-73.373255 | Meta | ***R.pictipes*** | Sylvatic |
| **29** | 4.29457,-73.373255 | Meta | ***P.geniculatus*** | Sylvatic |
| **30** | 4.29457,-73.373255 | Meta | ***P.geniculatus*** | Peridomestic |
| **31** | 4.29457,-73.373255 | Meta | ***P.geniculatus*** | Peridomestic |
| **32** | 4.29457,-73.373255 | Meta | ***P.geniculatus*** | Peridomestic |
| **33** | 4.29457,-73.373255 | Meta | ***P.geniculatus*** | Peridomestic |
| **34** | 4.29457,-73.373255 | Meta | ***P.geniculatus*** | Peridomestic |
| **35** | 4.29457,-73.373255 | Meta | ***P.geniculatus*** | Peridomestic |
| **36** | 4.29457,-73.373255 | Meta | ***P.geniculatus*** | Peridomestic |
| **37** | 4.29457,-73.373255 | Meta | ***P.geniculatus*** | Peridomestic |
| **38** | 4.29457,-73.373255 | Meta | ***P.geniculatus*** | Peridomestic |
| **39** | 4.29457,-73.373255 | Meta | ***P.geniculatus*** | Peridomestic |
| **40** | 4.29457,-73.373255 | Meta | ***P.geniculatus*** | Peridomestic |
| **41** | 4.29457,-73.373255 | Meta | ***P.geniculatus*** | Peridomestic |
| **42** | 4.29457,-73.373255 | Meta | ***P.geniculatus*** | Peridomestic |
| **43** | 4.29457,-73.373255 | Meta | ***P.geniculatus*** | Peridomestic |
| **Number** | **Coordinates** | **Location** | **Species** | **Ecotopes** |
| **44** | 4.29457,-73.373255 | Meta | ***P.geniculatus*** | Peridomestic |
| **45** | 4.29457,-73.373255 | Meta | ***P.geniculatus*** | Peridomestic |
| **46** | 8.491166,-76.678694 | Antioquía | **R.pallescens** | Sylvatic |
| **47** | 8.491166,-76.678694 | Antioquía | **R.pallescens** | Sylvatic |
| **48** | 8.491166,-76.678694 | Antioquía | **R.pallescens** | Sylvatic |
| **49** | 8.491166,-76.678694 | Antioquía | **R.pallescens** | Peridomestic |
| **50** | 8.491166,-76.678694 | Antioquía | **R.pallescens** | Domestic |
| **51** | 8.491166,-76.678694 | Antioquía | **R.pallescens** | Domestic |
| **52** | 8.710554,-76.530023 | Antioquía | **R.pallescens** | Sylvatic |
| **53** | 8.491166,-76.678694 | Antioquía | **R.pallescens** | Domestic |
| **54** | 8.491166,-76.678694 | Antioquía | **R.pallescens** | Domestic |
| **55** | 8.491166,-76.678694 | Antioquía | **R.pallescens** | Domestic |
| **56** | 8.491166,-76.678694 | Antioquía | **R.pallescens** | Domestic |
| **57** | 8.491166,-76.678694 | Antioquía | **R.pallescens** | Sylvatic |
| **58** | 8.491166,-76.678694 | Antioquía | **R.pallescens** | Sylvatic |
| **59** | 8.491166,-76.678694 | Antioquía | **R.pallescens** | Sylvatic |
| **60** | 8.491166,-76.678694 | Antioquía | **R.pallescens** | Sylvatic |
| **61** | 8.491166,-76.678694 | Antioquía | **R.pallescens** | Sylvatic |
| **62** | 8.491166,-76.678694 | Antioquía | **R.pallescens** | Sylvatic |
| **63** | 8.491166,-76.678694 | Antioquía | **R.pallescens** | Sylvatic |
| **64** | 8.491166,-76.678694 | Antioquía | **R.pallescens** | Sylvatic |
| **65** | 8.491166,-76.678694 | Antioquía | **R.pallescens** | Sylvatic |
| **66** | 8.491166,-76.678694 | Antioquía | **R.pallescens** | Sylvatic |
| **67** | 6.984055,-74.549545 | Antioquía | **R.pallescens** | Peridomestic |
| **68** | 8.491166,-76.678694 | Antioquía | **R.pallescens** | Sylvatic |
| **69** | 8.491166,-76.678694 | Antioquía | **R.pallescens** | Sylvatic |
| **70** | 8.491166,-76.678694 | Antioquía | **R.pallescens** | Sylvatic |
| **71** | 8.491166,-76.678694 | Antioquía | **R.pallescens** | Sylvatic |
| **72** | 8.710554,-76.530023 | Antioquía | **R.pallescens** | Sylvatic |
| **73** | 8.710554,-76.530023 | Antioquía | **R.pallescens** | Sylvatic |
| **74** | 8.491166,-76.678694 | Antioquía | ***T.dimidiata*** | Sylvatic |
| **75** | 5.879258,-71.908682 | Casanare | ***R.prolixus*** | Peridomestic |
| **76** | 5.879258,-71.908682 | Casanare | ***R.prolixus*** | Peridomestic |
| **77** | 5.879258,-71.908682 | Casanare | ***R.prolixus*** | Domestic |
| **78** | 11.066486,-72.737381 | Guajira | ***T. maculata*** | Peridomestic |
| **79** | 11.066486,-72.737381 | Guajira | ***T. maculata*** | Peridomestic |
| **80** | 11.066486,-72.737381 | Guajira | ***T. maculata*** | Peridomestic |
| **81** | 11.066486,-72.737381 | Guajira | ***T. maculata*** | Peridomestic |
| **82** | 11.066486,-72.737381 | Guajira | ***T. maculata*** | Peridomestic |
| **83** | 11.066486,-72.737381 | Guajira | ***T. maculata*** | Peridomestic |
| **84** | 8.972489,-73.165056 | Norte de santander | ***R.prolixus*** | No Date |
| **85** | 8.972489,-73.165056 | Norte de santander | ***R.prolixus*** | No Date |
| **86** | 8.972489,-73.165056 | Norte de santander | ***R.prolixus*** | No Date |
| **87** | 8.972489,-73.165056 | Norte de santander | ***R.prolixus*** | No Date |
| **Number** | **Coordinates** | **Location** | **Species** | **Ecotopes** |
| **88** | 8.972489,-73.165056 | Norte de santander | ***R.prolixus*** | No Date |
| **89** | 8.972489,-73.165056 | Norte de santander | ***R.prolixus*** | No Date |
| **90** | 8.972489,-73.165056 | Norte de santander | ***R.prolixus*** | No Date |
| **91** | 8.972489,-73.165056 | Norte de santander | ***R.prolixus*** | No Date |
| **92** | 8.64, -72.738056 | Norte de santander | ***R.prolixus*** | Peridomestic |
| **93** | 8.64, -72.738056 | Norte de santander | ***R.prolixus*** | Sylvatic |
| **94** | 8.64, -72.738056 | Norte de santander | ***R.prolixus*** | Peridomestic |
| **95** | 8.64, -72.738056 | Norte de santander | ***R.prolixus*** | Peridomestic |
| **96** | 8.64, -72.738056 | Norte de santander | ***R.prolixus*** | Peridomestic |
| **97** | 8.64, -72.738056 | Norte de santander | ***R.prolixus*** | Domestic |
| **98** | 8.64, -72.738056 | Norte de santander | ***R.prolixus*** | Peridomestic |
| **99** | 8.64, -72.738056 | Norte de santander | ***R.prolixus*** | Peridomestic |
| **100** | 8.64, -72.738056 | Norte de santander | ***R.prolixus*** | Domestic |
| **101** | 8.64, -72.738056 | Norte de santander | ***R.prolixus*** | No Date |
| **102** | 8.64, -72.738056 | Norte de santander | ***R.prolixus*** | No Date |
| **103** | 8.64, -72.738056 | Norte de santander | ***R.prolixus*** | No Date |
| **104** | 10.600085,-73.516405 | Cesar | ***R.prolixus*** | Peridomestic |
| **105** | 10.34291,-73.4575 | Cesar | ***R.prolixus*** | Peridomestic |
| **106** | 10.34291,-73.4575 | Cesar | ***R.prolixus*** | Peridomestic |
| **107** | 10.34291,-73.4575 | Cesar | ***R.prolixus*** | Peridomestic |
| **108** | 10.34291,-73.4575 | Cesar | ***R.prolixus*** | Domestic |
| **109** | 10.34291,-73.4575 | Cesar | ***R.prolixus*** | Domestic |
| **110** | 10.34291,-73.4575 | Cesar | ***R.prolixus*** | Peridomestic |
| **111** | 10.34291,-73.4575 | Cesar | ***R.prolixus*** | Domestic |
| **112** | 10.34291,-73.4575 | Cesar | ***R.prolixus*** | Peridomestic |
| **113** | 10.34291,-73.4575 | Cesar | ***R.prolixus*** | Domestic |
| **114** | 10.34291,-73.4575 | Cesar | ***R.prolixus*** | Domestic |
| **115** | 10.34291,-73.4575 | Cesar | ***R.prolixus*** | Domestic |
| **116** | 10.34291,-73.4575 | Cesar | ***R.prolixus*** | Peridomestic |
| **117** | 10.34291,-73.4575 | Cesar | ***R.prolixus*** | Peridomestic |
| **118** | 10.34291,-73.4575 | Cesar | ***R.prolixus*** | Peridomestic |
| **119** | 10.34291,-73.4575 | Cesar | ***R.prolixus*** | Domestic |
| **120** | 10.34291,-73.4575 | Cesar | ***R.prolixus*** | Domestic |
| **121** | 10.34291,-73.4575 | Cesar | ***R.prolixus*** | Domestic |
| **122** | 10.34291,-73.4575 | Cesar | ***R.prolixus*** | Domestic |
| **123** | 10.34291,-73.4575 | Cesar | ***R.prolixus*** | Peridomestic |
| **124** | 10.34291,-73.4575 | Cesar | ***R.prolixus*** | Peridomestic |
| **125** | 10.34291,-73.4575 | Cesar | ***R.prolixus*** | Peridomestic |
| **126** | 10.34291,-73.4575 | Cesar | ***R.prolixus*** | Domestic |
| **127** | 10.34291,-73.4575 | Cesar | ***R.prolixus*** | Peridomestic |
| **128** | 10.34291,-73.4575 | Cesar | ***R.prolixus*** | Domestic |
| **129** | 10.34291,-73.4575 | Cesar | ***R.prolixus*** | Peridomestic |
| **130** | 10.34291,-73.4575 | Cesar | ***R.prolixus*** | Peridomestic |
| **131** | 10.34291,-73.4575 | Cesar | ***R.prolixus*** | Domestic |
| **Number** | **Coordinates** | **Location** | **Species** | **Ecotopes** |
| **132** | 10.34291,-73.4575 | Cesar | ***R.prolixus*** | Peridomestic |
| **133** | 10.34291,-73.4575 | Cesar | ***R.prolixus*** | Peridomestic |
| **134** | 10.34291,-73.4575 | Cesar | ***R.prolixus*** | Domestic |
| **135** | 10.34291,-73.4575 | Cesar | ***R.prolixus*** | Domestic |
| **136** | 10.34291,-73.4575 | Cesar | ***R.prolixus*** | Domestic |
| **137** | 10.34291,-73.4575 | Cesar | ***R.prolixus*** | Peridomestic |
| **138** | 10.34291,-73.4575 | Cesar | ***R.prolixus*** | Domestic |
| **139** | 10.34291,-73.4575 | Cesar | ***T.dimidiata*** | Domestic |
| **140** | 10.469876,-73.254253 | Cesar | **R.pallescens** | Sylvatic |
| **141** | 10.469876,-73.254253 | Cesar | **R.pallescens** | Sylvatic |
| **142** | 10.469876,-73.254253 | Cesar | **R.pallescens** | Sylvatic |
| **143** | 10.469876,-73.254253 | Cesar | **R.pallescens** | Sylvatic |
| **144** | 10.469876,-73.254253 | Cesar | **R.pallescens** | Sylvatic |
| **145** | 10.469876,-73.254253 | Cesar | **R.pallescens** | Sylvatic |
| **146** | 10.469876,-73.254253 | Cesar | **R.pallescens** | Sylvatic |
| **147** | 10.469876,-73.254253 | Cesar | **R.pallescens** | Sylvatic |
| **148** | 10.469876,-73.254253 | Cesar | **R.pallescens** | Sylvatic |
| **149** | 10.469876,-73.254253 | Cesar | ***T. maculata*** | Sylvatic |
| **150** | 10.812875,-73.099985 | Guajira | ***R.prolixus*** | Domestic |
| **151** | 10.812875,-73.099985 | Guajira | ***R.prolixus*** | Domestic |
| **152** | 10.812875,-73.099985 | Guajira | ***R.prolixus*** | Peridomestic |
| **153** | 10.812875,-73.099985 | Guajira | ***R.prolixus*** | No Date |
| **154** | 10.812875,-73.099985 | Guajira | ***R.prolixus*** | No Date |
| **155** | 10.812875,-73.099985 | Guajira | ***R.prolixus*** | Domestic |
| **156** | 10.812875,-73.099985 | Guajira | ***R.prolixus*** | Domestic |
| **157** | 10.812875,-73.099985 | Guajira | ***R.prolixus*** | Domestic |
| **158** | 10.812875,-73.099985 | Guajira | ***R.prolixus*** | Domestic |
| **159** | 10.812875,-73.099985 | Guajira | ***R.prolixus*** | Domestic |
| **160** | 10.812875,-73.099985 | Guajira | ***R.prolixus*** | Domestic |
| **161** | 10.812875,-73.099985 | Guajira | ***R.prolixus*** | Peridomestic |
| **162** | 10.812875,-73.099985 | Guajira | ***R.prolixus*** | Domestic |
| **163** | 10.812875,-73.099985 | Guajira | ***R.prolixus*** | Peridomestic |
| **164** | 10.34291,-73.4575 | Cesar | ***R.prolixus*** | Peridomestic |
| **165** | 11.066486,-72.737381 | Guajira | ***T. maculata*** | Peridomestic |
| **166** | 11.49821,-72.98294 | Guajira | ***T. maculata*** | No Data |
| **167** | 5.879258,-71.908682 | Casanare | ***R.prolixus*** | No Data |
| **168** | 5.879258,-71.908682 | Casanare | ***R.prolixus*** | No Data |
| **169** | 2.386669, -75.5461 | Huila | ***T.dimidiata*** | No Data |
| **170** | 2.386669, -75.5461 | Huila | ***T.dimidiata*** | No Data |
| **171** | 4.29457,-73.373255 | Meta | ***P.geniculatus*** | Domestic |
| **172** | 4.29457,-73.373255 | Meta | ***P.geniculatus*** | Domestic |
| **173** | 4.29457,-73.373255 | Meta | ***P.geniculatus*** | Domestic |
| **174** | 4.29457,-73.373255 | Meta | ***P.geniculatus*** | Domestic |
| **175** | 4.29457,-73.373255 | Meta | ***P.geniculatus*** | Domestic |
| **Number** | **Coordinates** | **Location** | **Species** | **Ecotopes** |
| **176** | 4.29457,-73.373255 | Meta | ***P.geniculatus*** | Domestic |
| **177** | 4.29457,-73.373255 | Meta | ***P.geniculatus*** | Domestic |
| **178** | 4.29457,-73.373255 | Meta | ***P.geniculatus*** | Domestic |
| **179** | 4.29457,-73.373255 | Meta | ***P.geniculatus*** | Domestic |
| **180** | 4.29457,-73.373255 | Meta | ***P.geniculatus*** | Domestic |
| **181** | 4.29457,-73.373255 | Meta | ***P.geniculatus*** | Domestic |
| **182** | 4.29457,-73.373255 | Meta | ***P.geniculatus*** | Domestic |
| **183** | 4.29457,-73.373255 | Meta | ***R.pictipes*** | Peridomestic |
| **184** | 4.29457,-73.373255 | Meta | ***R.prolixus*** | Peridomestic |
| **185** | 4.29457,-73.373255 | Meta | ***R.pictipes*** | Peridomestic |
| **186** | 4.29457,-73.373255 | Meta | ***R.pictipes*** | Domestic |
| **187** | 4.31417,-72.0825 | Meta | ***R.prolixus*** | Domestic |
| **188** | 4.29457,-73.373255 | Meta | ***P.geniculatus*** | Domestic |
| **189** | 4.29457,-73.373255 | Meta | ***P.geniculatus*** | Domestic |
| **190** | 4.29457,-73.373255 | Meta | ***P.geniculatus*** | Domestic |
| **191** | 4.29457,-73.373255 | Meta | ***P.geniculatus*** | Domestic |
| **192** | 4.29457,-73.373255 | Meta | ***P.geniculatus*** | Domestic |
| **193** | 4.29457,-73.373255 | Meta | ***P.geniculatus*** | Domestic |
| **194** | 4.29457,-73.373255 | Meta | ***P.geniculatus*** | Domestic |
| **195** | 4.29457,-73.373255 | Meta | ***P.geniculatus*** | Domestic |
| **196** | 4.29457,-73.373255 | Meta | ***P.geniculatus*** | Domestic |
| **197** | 4.29457,-73.373255 | Meta | ***P.geniculatus*** | Domestic |
| **198** | 4.29457,-73.373255 | Meta | ***P.geniculatus*** | Domestic |
| **199** | 4.29457,-73.373255 | Meta | ***P.geniculatus*** | Domestic |
| **200** | 4.29457,-73.373255 | Meta | ***P.geniculatus*** | Domestic |
| **201** | 4.29457,-73.373255 | Meta | ***P.geniculatus*** | Domestic |
| **202** | 4.29457,-73.373255 | Meta | ***P.geniculatus*** | Domestic |
| **203** | 4.29457,-73.373255 | Meta | ***P.geniculatus*** | Domestic |
| **204** | 4.32194, -72.455 | Meta | ***R.pictipes*** | Domestic |
| **205** | 4.29457,-73.373255 | Meta | ***P.geniculatus*** | Domestic |
| **206** | 4.29457,-73.373255 | Meta | ***P.geniculatus*** | Domestic |
| **207** | 4.29457,-73.373255 | Meta | ***P.geniculatus*** | Domestic |
| **208** | 4.29457,-73.373255 | Meta | ***P.geniculatus*** | Domestic |
| **209** | 4.29457,-73.373255 | Meta | ***P.geniculatus*** | Domestic |
| **210** | 4.29457,-73.373255 | Meta | ***P.geniculatus*** | Domestic |
| **211** | 4.29457,-73.373255 | Meta | ***P.geniculatus*** | Domestic |
| **212** | 4.29457,-73.373255 | Meta | ***P.geniculatus*** | Domestic |
| **213** | 4.29457,-73.373255 | Meta | ***P.geniculatus*** | Domestic |
| **214** | 4.29457,-73.373255 | Meta | ***P.geniculatus*** | Peridomestic |
| **215** | 4.29457,-73.373255 | Meta | ***P.geniculatus*** | Domestic |
| **216** | 4.29457,-73.373255 | Meta | ***P.geniculatus*** | Domestic |
| **217** | 4.29457,-73.373255 | Meta | ***P.geniculatus*** | Domestic |
| **218** | 4.29457,-73.373255 | Meta | ***P.geniculatus*** | Domestic |
| **219** | 4.29457,-73.373255 | Meta | ***P.geniculatus*** | Domestic |
| **Number** | **Coordinates** | **Location** | **Species** | **Ecotopes** |
| **220** | 4.29457,-73.373255 | Meta | ***P.geniculatus*** | Domestic |
| **221** | 4.29457,-73.373255 | Meta | ***R.pictipes*** | Domestic |
| **222** | 4.29457,-73.373255 | Meta | ***P.geniculatus*** | Domestic |
| **223** | 4.29457,-73.373255 | Meta | ***P.geniculatus*** | Peridomestic |
| **224** | 4.29457,-73.373255 | Meta | ***P.geniculatus*** | Peridomestic |
| **225** | 4.29457,-73.373255 | Meta | ***P.geniculatus*** | Peridomestic |
| **226** | 4.29457,-73.373255 | Meta | ***P.geniculatus*** | Peridomestic |
| **227** | 4.29457,-73.373255 | Meta | ***P.geniculatus*** | Peridomestic |
| **228** | 4.29457,-73.373255 | Meta | ***P.geniculatus*** | Peridomestic |
| **229** | 4.29457,-73.373255 | Meta | ***P.geniculatus*** | Peridomestic |
| **230** | 4.29457,-73.373255 | Meta | ***P.geniculatus*** | Peridomestic |
| **231** | 4.29457,-73.373255 | Meta | ***P.geniculatus*** | Domestic |
| **232** | 4.29457,-73.373255 | Meta | ***P.geniculatus*** | Peridomestic |
| **233** | 4.29457,-73.373255 | Meta | ***P.geniculatus*** | Peridomestic |
| **234** | 4.29457,-73.373255 | Meta | ***P.geniculatus*** | Peridomestic |
| **235** | 4.29457,-73.373255 | Meta | ***P.geniculatus*** | Peridomestic |
| **236** | 4.29457,-73.373255 | Meta | ***P.geniculatus*** | Peridomestic |
| **237** | 4.29457,-73.373255 | Meta | ***P.geniculatus*** | Peridomestic |
| **238** | 4.29457,-73.373255 | Meta | ***P.geniculatus*** | Peridomestic |
| **239** | 4.29457,-73.373255 | Meta | ***P.geniculatus*** | Peridomestic |
| **240** | 4.29457,-73.373255 | Meta | ***P.geniculatus*** | Peridomestic |
| **241** | 4.29457,-73.373255 | Meta | ***P.geniculatus*** | Peridomestic |
| **242** | 4.29457,-73.373255 | Meta | ***P.geniculatus*** | Peridomestic |
| **243** | 4.29457,-73.373255 | Meta | ***P.geniculatus*** | Peridomestic |
| **244** | 4.29457,-73.373255 | Meta | ***P.geniculatus*** | Domestic |
| **245** | 4.29457,-73.373255 | Meta | ***P.geniculatus*** | Domestic |
