# Supplementary material for: Untangling the transmission dynamics of primary and secondary vectors of Trypanosoma cruzi in Colombia: parasite infection, feeding sources and discrete typing units
Source: Parasit Vectors. 2016 Dec 1;9:620. doi: 10.1186/s13071-016-1907-5 (PMC5131512; doi:10.1186/s13071-016-1907-5)
Supplement: Additional file 2: Table S2: — T. cruzi infection rates in the triatomine species studied. (DOCX 16 kb) [file 13071_2016_1907_MOESM2_ESM.docx]

**Table S2. Positivity of *T. cruzi* in the triatomine species studied**

| *Species* | *T. cruzi (+)*  *n* | *T. cruzi (-)*  *n* | *Total*  *n (%)* | *X^2^* | *G-test* |
| --- | --- | --- | --- | --- | --- |
| *P. geniculatus* | *60* | *25* | *85* | ***1.835 x 10 ^-7^*** | ***0.000184*** |
| *R. prolixus* | *42* | *35* | *77* | *0.3336* | *0.4939* |
| *T. maculata* | *23* | *11* | *44* | ***0.0163*** | *0.056* |
| *R. pallescens* | 17 | 20 | 37 | *0.6419* | *0.7422* |
| *R. pictipes* | *7* | *1* | *8* | ***0.01242*** | 0.06643 |
| *T. dimidiata* | *1* | *3* | *4* | *0.4795* | *0.6152* |
| *Total* | *150* | *95* | *245* |  |  |

*FD: 5,* ***X^2^ with Monte Carlo adjustment/10000 simulations*** *=13.35 p= 0.0171,* ***Gtest****=13.33, p=0, 0175*
