# Supplementary material for: Untangling the transmission dynamics of primary and secondary vectors of Trypanosoma cruzi in Colombia: parasite infection, feeding sources and discrete typing units
Source: Parasit Vectors. 2016 Dec 1;9:620. doi: 10.1186/s13071-016-1907-5 (PMC5131512; doi:10.1186/s13071-016-1907-5)
Supplement: Additional file 3: Table S3: — Pairwise G-test (T. cruzi infection rates by species). (DOCX 13 kb) [file 13071_2016_1907_MOESM3_ESM.docx]

**Table S3. Pairwise G-test (*T.cruzi* positivity by species)**

|  | ***P. geniculatus*** | ***R. pallescens*** | ***R. pictipes*** | ***R. prolixus*** | ***T. maculata*** |
| --- | --- | --- | --- | --- | --- |
| ***R. pallescens*** | ***0.010*** | *-* | *-* | *-* | *-* |
| ***R. pictipes*** | *0.273* | ***0.024*** | *-* | *-* | *-* |
| ***R. prolixus*** | ***0.034*** | *0.390* | *0.054* | *-* | *-* |
| ***T. maculata*** | *0.753* | *0.064* | *0.233* | *0.193* | *-* |
| ***T.dimidiata*** | *0.067* | *0.410* | ***0.029*** | *0.241* | *0.100* |
