# Supplementary material for: Untangling the transmission dynamics of primary and secondary vectors of Trypanosoma cruzi in Colombia: parasite infection, feeding sources and discrete typing units
Source: Parasit Vectors. 2016 Dec 1;9:620. doi: 10.1186/s13071-016-1907-5 (PMC5131512; doi:10.1186/s13071-016-1907-5)
Supplement: Additional file 4: Table S4: — Pairwise G-test (Species vs Ecotopes). (DOCX 13 kb) [file 13071_2016_1907_MOESM4_ESM.docx]

**Table S4. Pairwise G-test (Species vs. Ecotopes)**

|  | ***P. geniculatus*** | ***R.pallescens*** | ***R. pictipes*** | ***R. prolixus*** |
| --- | --- | --- | --- | --- |
| ***R.pallescens*** | ***< 2 x 10 ^-16^*** | *-* | *-* | *-* |
| ***R. pictipes*** | ***0.0030*** | *0.0740* | *-* | *-* |
| ***R.prolixus*** | *0.7043* | ***< 2 x 10 ^-16^*** | ***0.00654*** | *-* |
| ***T. maculata*** | ***0.0009*** | ***1.7 x 10 ^-10^*** | ***0.04982*** | ***0.01330*** |
