# Supplementary material for: Untangling the transmission dynamics of primary and secondary vectors of Trypanosoma cruzi in Colombia: parasite infection, feeding sources and discrete typing units
Source: Parasit Vectors. 2016 Dec 1;9:620. doi: 10.1186/s13071-016-1907-5 (PMC5131512; doi:10.1186/s13071-016-1907-5)
Supplement: Additional file 5: Table S5: — Pairwise G-test (T. cruzi infection rates vs Geographical location). (DOCX 14 kb) [file 13071_2016_1907_MOESM5_ESM.docx]

**Table S5. Pairwise G-test (*T. cruzi* positivity vs. Geographical location)**

|  | ***Antioquia*** | ***Casanare*** | ***Cesar*** | ***Guajira*** | ***Huila*** | ***Meta*** |
| --- | --- | --- | --- | --- | --- | --- |
| ***Casanare*** | *0.4401* | *-* | *-* | *-* | *-* | *-* |
| ***Cesar*** | *0.5202* | *0.6370* | *-* | *-* | *-* | *-* |
| ***Guajira*** | ***0.0129*** | *0.6450* | ***0.0347*** | *-* | *-* | *-* |
| ***Huila*** | *0.8123* | *0.8097* | *0.9765* | *0.5592* | *-* | *-* |
| ***Meta*** | ***0.0024*** | *0.5532* | ***0.0058*** | *0.7637* | *0.5033* | *-* |
| ***Norte de Santander*** | *0.8014* | *0.8014* | *0.7677* | *0.0530* | *0.8925* | ***0.0196*** |
